# The association between alcohol consumption and intimate partner violence in young male perpetrators in Mwanza, Tanzania: a cross-sectional study

**DOI:** 10.1080/16549716.2023.2185967

**Published:** 2023-03-16

**Authors:** Olena Shubina, Gerry Mshana, Simon Sichalwe, Donati Malibwa, Neema Mosha, Ramadhan Hashim, Fauzia Nahay, Philip Ayieko, Saidi Kapiga, Heidi Stöckl

**Affiliations:** a Institute for Medical Information Processing, Biometry and Epidemiology, Medical Faculty, Ludwig-Maximilians-University, Munich, Germany; b Pettenkofer School of Public Health, Munich, Germany; c National Institute for Medical Research, Mwanza, Tanzania; d Mwanza Intervention Trials Unit, Mwanza, Tanzania; e Department of Infectious Epidemiology, London School of Hygiene and Tropical Medicine, London, UK; f Department of Global Health and Development, London School of Hygiene and Tropical Medicine, UK

**Keywords:** Intimate partner violence, alcohol use, AUDIT, violence against women and girls, young men

## Abstract

**Background:**

Although alcohol consumption is a well-known risk factor for intimate partner violence (IPV) perpetration, few studies have been conducted among young males in low- and middle-income countries. Alcohol consumption and IPV are both complex phenomena, whose association requires more in-depth exploration regarding drinking patterns and the alcohol-related manifestation of five different forms of IPV.

**Objective:**

In this study, we sought to explore the relationship between alcohol use and IPV in young Tanzanian men and to identify differences in the magnitude of past-year IPV perpetration among alcohol drinkers and abstainers. Furthermore, we aimed to assess the association between various drinking patterns with the perpetration of different forms of IPV.

**Methods:**

A cross-sectional survey of 1002 young males residing in Mwanza, Tanzania, was conducted in 2021–2022. Data on alcohol consumption were collected using the alcohol use disorder identification test. IPV perpetration was assessed using an index total of 19 items on acts of physical, sexual, economic, emotional abuse, and controlling behaviour. Logistic regression models were conducted to estimate the relationship between alcohol use and the perpetration of each form of IPV.

**Results:**

Among partnered respondents currently consuming alcohol (*n* = 189, 18.8%), the most and the least prevalent IPV forms in the past 12 months were controlling behaviour (84.1%) and physical IPV (25.4%), respectively. Those reporting recent alcohol consumption reported higher rates of all forms of past-year IPV perpetration compared to abstainers. While no form of IPV was associated with low-risk consumption versus abstention, all forms of IPV were associated with hazardous drinking.

**Conclusion:**

Young men who drink alcohol, especially those drinking hazardously, are also more likely to report perpetrating IPV. An understanding of the different drinking patterns and manifestations of forms of IPV can contribute to better-tailored alcohol-related interventions and has the potential to improve young adults’ health and reduce IPV perpetration.

## Introduction

Male-perpetrated intimate partner violence (IPV) is recognised as a serious public health issue worldwide [1]. IPV manifests in different forms including physical, sexual, emotional, economic, and controlling behaviours, which often co-exist together [2,3].

IPV is pervasive throughout various settings, social positions, and hetero- and same-sex relationships and has been recognised as a gendered issue [4]. It is estimated that globally around 30% of the women have experienced at least one form of IPV during their lifetime [5]. Additionally, violence in relationships is a recognised risk factor for homicide. At least one of the three cases of women being murdered worldwide is perpetrated by a current or former male intimate partner [6,7]. IPV is associated with a range of adverse health outcomes, such as poor physical and mental health, injuries, substance use, depression, post-traumatic stress disorder, anxiety, and suicide [8–10]. While there is substantial evidence on the risk and protective factors for women experiencing IPV, less is known about the factors associated with men’s perpetration of IPV, examined from the male perspective.

Alcohol is a well-documented and commonly cited modifiable risk factor for male-perpetrated IPV [11]. In particular, frequent and hazardous alcohol use is found to be associated with an elevated risk of IPV perpetration [12]. Numerous explanations exist for the mechanisms underlying the association between alcohol consumption and the perpetration of IPV. First, alcohol consumption affects cognitive functioning. In the context of an intimate relationship, this may result in ineffective problem-solving, distorted perception of the partner’s actions and violent reactions to them [13]. Second, alcohol can facilitate aggressive behaviours in both men and women; however, the effect is stronger in men [14,15]. Alcohol-related aggression is also explained by the I^3^ theory, based on the premise that three processes cause aggression: instigation, impellance, and inhibition [16]. Third, alcohol creates a high potential for conflict due to greater inhibition and control over emotions as well as higher levels of aggression, which can result in a marital conflict between partners at the moment of intoxication, over the amount of consumed alcohol and because of unfulfilled expectations because of intoxication [17]. On the other hand, men may use alcohol to cope with existing conflict in the relationship [17]. Despite these existing theories and pathways between alcohol and IPV, the nature of its relationship remains complex and unclear due to the presence of other mediating factors that are very diverse, interwoven, and operate on different levels [18]. The ecological approach suggested by Heise [19] illustrates IPV as a multifaceted phenomenon that results from an interplay of personal, situational, and socio-cultural factors. One of the recent contributions to the IPV research targeting the perpetrator perspective in the sub-Saharan setting was a finding that food insecurity, which is used as a proxy for poverty, was associated with doubled odds of perpetration of IPV, with food insecurity itself being associated with alcohol abuse [20]. A study in Benin, for example, identified alcohol consumption as a coping strategy for food insecurity and an exacerbator of tensions caused by it [21].

The majority of evidence exploring the alcohol–IPV link comes from high-income countries, and little research has been conducted in sub-Saharan Africa. A systematic review on the global prevalence of IPV shows that the central region of sub-Saharan Africa reports the highest levels (65.64%) of IPV in ever-partnered women [22]. Few studies on this subject have been conducted in Tanzania, despite high levels of IPV reported from the country. For example, the Tanzanian Demographic Health Survey (DHS) 2015/2016 reported that 50% of the ever-married women have experienced physical, sexual, or emotional IPV in their lifetime [23]. According to the Global Status Report on Alcohol and Health by the WHO [24], Africa is facing a growing burden of problematic alcohol use. The average alcohol consumption in the African continent is 13% higher than the global average, especially among young people [18]. In Tanzania, 52% of the young people between 15 and 24 years reported ever using alcohol and 26% used it in the past 12 months. This age group is also highly exposed to alcohol advertisements and faces widespread accessibility in Northern Tanzania [25,26]. In sub-Saharan Africa, alcohol use also shows striking gender patterns. According to Francis et al., men were by far more likely to report currently drinking alcohol, to engage in heavy drinking, and to screen positive for alcohol use disorders (AUDs) compared to women [27].

Evidence targeting the perpetrator’s perspective on IPV is scarce. The majority of studies in low- and middle-income countries focuses on women’s experience of IPV. As they are often based on the DHS, they capture the whole range of women of reproductive age, aged 15–49 years, limiting the ability to conduct focused research on young people based on small sample sizes in this age group [23]. Alongside alcohol, being male and of young age are other major contributors to IPV at the individual level, with IPV perpetration reaching its peak in the early twenties among young men [28]. Young adults are more likely to engage in high-risk behaviours such as substance abuse and hazardous drinking, leading to AUDs [26]. These interactions of risk factors demonstrate young men’s increased vulnerability to IPV perpetration.

It is crucial to understand the association between young men’s alcohol use and the manifestation of different forms of IPV to be able to develop tailored interventions for alcohol-related IPV reduction. Research targeting particularly vulnerable groups for IPV such аs young men should be prioritised not only to prevent IPV perpetration, but also to improve young men’s health.

The aim of this study was to identify differences in the magnitude of IPV perpetration among young men who consume alcohol versus abstainers. Additionally, we aimed to assess how different drinking patterns were associated with five different forms of IPV. Although there are numerous studies observing the linkage between alcohol consumption and IPV, the majority of this research focused on traditional forms of IPV such as physical, sexual, and emotional abuse. More differentiated data on the association between alcohol use among young men and all five forms of IPV perpetration by men are needed.

## Methods

### Study design and setting

We conducted a cross-sectional representative household survey of young men in the city of Mwanza, northern Tanzania, from June 2021 to April 2022.

### Sample

A team of specially trained research assistants recruited participants according to three eligibility criteria: (a) aged 18–24 years, (b) male sex, and (c) residing in Mwanza. Using OpenEpi for the power calculation, we estimated a sample size of 1000 participants based on 24 clusters of approximately 20 men in each cluster. The estimated past-year prevalence of IPV perpetration was 28%, based on the reports of women in a MAISHA survey conducted in Mwanza [29]. From two districts, Nyamagana and Ilemela, we randomly selected six wards (local administrative units) from both densely and sparsely populated wards. From each ward, four streets were included, totalling 24 streets. GPS maps using QGIS software were used to identify 120 visiting points in each street. At each point, the two houses closest to the point were visited to inquire if young men aged 18–24 years resided there. A total of 2976 points were visited, resulting in the identification of 1065 young men, of whom 1002 were eligible, consented to participate in the study, and participated. If more than one young man in the chosen household met our inclusion criteria, a random selection was conducted by having a family member randomly pick a folded paper on which the young men’s names were written. The procedure was done transparently to ensure that all potential candidates had equal chances of being selected. The informed consent sheet, as well as the questionnaire, was provided in Swahili, the widely spoken national language.

To protect the safety and confidentiality of the respondents, interviewers conducted interviews in a private space, and no personal identification was collected. The interview was initially face-to-face to build rapport, and the researcher entered the responses to questions into a tablet. For the questionnaire section assessing sensitive questions on the perpetration of IPV, the tablet, programmed for use as ACASI, was handed to the participants for self-completion. Data were uploaded daily by the data manager and checked for inconsistencies.

Of the 1002 participants, only 754 who had ever been in a relationship were included in this analysis, based on their answer to the question: ‘Have you ever had a relationship where you were boyfriend-girlfriend, married or more casually dating someone?’

Ethical approval for this study was obtained from the ethics committee of the National Institute for Medical Research (NIMR/HQ/R.8a/Vol.IX/2991), the Ludwig-Maximilians-University Munich (Nr. 21–0508), and the London School of Hygiene and Tropical Medicine (16121).

#### IPV perpetration

Men’s past 12-month perpetration of IPV captured five different forms of IPV, using 19 acts capturing physical, sexual, economic, and emotional abuse, as well as controlling behaviours. They included questions on physical violent behaviours, such as hitting and kicking, forced sexual acts, controlling partner’s finances and financial decisions, and controlling behaviours, such as prohibiting visiting friends and family, as well as humiliating, intimidating, and threatening actions towards their intimate partner. A binary variable for each form of IPV was generated if the respondent answered positively to at least one act of the specific form of IPV. The IPV questions were adopted from the Sonke CHANGE Trial Questionnaire, CoVAC Adolescent Questionnaire, and IMAGES questionnaire [30,31].

#### Alcohol consumption

To assess alcohol consumption, drinking patterns, and alcohol-related problems, we used the validated 10-itеm Alcohol Usе Disordеrs Idеntification Test (AUDIT) developed by the WHO [32]. The questionnaire assesses three dоmains: past year alcоhоl intake, dependence symptoms, and alcohol-related problems [33]. The total AUDIT score ranges from 0 to 40 points and was analysed as an ordinal variable with five recommended cut-off scores that were applied: abstainer (AUDIT 0), low-risk drinking (AUDIT 1–7), hazardous drinking (AUDIT 8–15), problematic drinking (AUDIT 16–19), and possible alcohol dependence (AUDIT 20–40). Any cut-off score of ≥8 is considered an indication of AUDs [33].

#### Covariates

**Figure 1.**
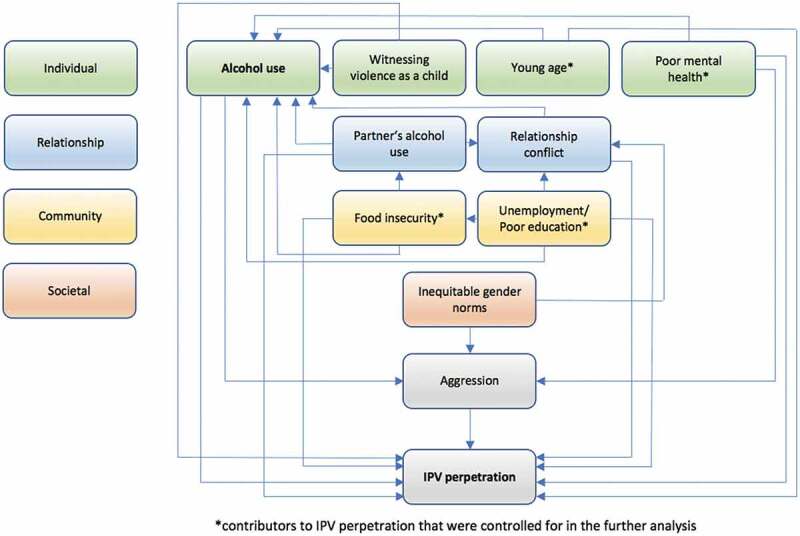
Framework linking alcohol use and other contributors to IPV on different levels.

As outlined in our conceptual framework (Figure 1), we identified variables that could be associated with IPV and alcohol use [19,20]. We also identified five covariates associated with IPV that were assessed in our questionnaire. These include age as a continuous variable ranging from 18 to 24. Education was coded in four categories: (a) never went to school/primary incomplete, (b) primary education complete, (c) secondary education complete, and (d) college training/university. Employment in the past 12 months was coded as a nominal variable (yes/no) and marital status as married or unmarried. Food insecurity was measured by four questions from the Household Food Insecurity Access Scale 2007. A nominal variable was created, coded as 1 if participants reported at least one episode of food insecurity in the past 12 months and coded as 0 if they did not report any of them. The Patient Health Questionnaire PHQ-9 tool was used to assess participants’ mental health. Symptoms of depression were considered minimal with a score below five, mild with a score between 5 and 9, moderate with a score between 10 and 14, moderately severe with a score between 15 and 19, and severe with a score of 20 and above [34].

### Statistical analysis

The analysis was conducted using Stata, version 17.0. Descriptive statistics were undertaken to describe the sample characteristics using percentages. We assessed the internal consistency of the scales by calculating Cronbach’s alpha (α). A chi-squared test was performed to assess the relationship between different forms of IPV perpetration, past-year alcohol consumption, drinking patterns, and other categorical covariates. To measure the association between IPV and recent alcohol consumption, five separate logistic regression models, one for each form of IPV, were conducted. Socio-demographic factors and other covariates associated with the perpetration of IPV as defined by the conceptual framework (Figure 1) were controlled for in all five logistic regression models. The models were adjusted for the clustering effect within the 24 sampled streets. The odds ratios (ORs), adjusted ORs (AORs), and α values presented, and a significant association was considered below 0.05. There were no missing values for the selected variables, only structurally missing data due to skipped follow-up questions that were not applicable to the respondents.

## Results

A total of 754 young men reported being in a relationship in the past 12 months, with a median age of 21 years. Nearly half of the participants (*n* = 347, 49.6%) did not complete post-secondary education. Only a small proportion of men were married (*n* = 64, 8.5%). Nearly a quarter of the participants (*n* = 181, 24.0%) were unemployed, while more than half reported experiencing food insecurity in the past 12 months (*n* = 419, 55.6%). Overall, 32% (*n* = 241) of the young men reported mild symptoms of depression, 7.8% (*n* = 59) moderate, and 1.6% (*n* = 8) severe levels of depression.

One-quarter of the study population reported past-year alcohol consumption (*n* = 189, 25.1%). According to the AUDIT, 12.7% (*n* = 96) were considered low-risk alcohol consumers, 9.0% (*n* = 68) to be engaged in hazardous drinking, 1.5% (*n* = 11) to have problematic drinking habits, and 1.9% (*n* = 14) to have a high dependence on alcohol. 165% (*n* = 124) of the respondents reported to perpetrate at least one form of IPV. The most common form of IPV perpetration was controlling behaviour (*n* = 599, 79.4%) followed by economic abuse (*n* = 357, 47.4%). Physical abuse was the least frequent type of IPV to be reported (*n* = 176, 23.3%).

The distribution of IPV prevalence according to participant’s AUDIT score is shown in Table 1. It illustrates that the prevalence of all forms of IPV increased among men considered to be at low risk of drinking compared to abstainers and increased even further among those considered hazardous drinkers. Overall, participants who reported past-year alcohol consumption also reported higher rates of IPV perpetration of all forms compared to abstainers: financial abuse 40.2% versus 27.4%, emotional abuse 57.1% versus 44.1%, physical abuse 25.4% versus 13.5%, sexual abuse 31.2% versus 20.7%, and controlling behaviours 84.1% versus 77.9%. The patterns among those with problematic drinking habits and those with possible dependence varied across forms of IPV. Cronbach’s α for all items measuring physical IPV was 0.72, the value for sexual IPV was α = 0.83, for emotional IPV α = 0.80, for financial IPV α = 0.71, and for controlling behaviours α = 0.77, yielding the overall good internal consistency of the interview questions measuring IPV.

**Table 1.** Prevalence of men’s perpetration of different forms of IPV by drinking patterns (*N* = 754).

Prevalence of IPV perpetration by drinking patterns/AUDIT score (n%)						
	Abstainers	Low-risk drinking	Hazardous drinking	Problematic drinking	Possible dependence	
Past-year IPV perpetration type	*n* = 565 (74.5)	*n* = 96 (12.7)	*n* = 68 (9.0)	*n* = 11 (1.5)	*n* = 14 (1.9)	*p*-value
Physical	13.45 (76)	14.58 (14)	38.24 (26)	36.36 (4)	28.57 (4)	<0.001*
Sexual	117 (20.7)	22 (22.9)	25 (36.8)	4 (36.4)	8 (57.1) (8)	0.001*
Financial	155 (27.4)	33 (34.4)	30 (44.1)	4 (36.4)	9 (64.3)	0.002*
Emotional	249 (44.1)	45 (46.9)	49 (72.1)	5 (45.5)	9 (64.3)	<0.001*
Controlling behavior	440 (77.98)	77 (80.2)	60 (88.2)	9 (81.8)	13 (92.9)	0.224

*Chi-squared test significance <0.05.

Overall, alcohol use was significantly associated with physical (<0.001), sexual (*p* = 0.001), financial (*p* = 0.002), and emotional IPV (<0.001), but not with controlling behaviours (*p* = 0.224).

In the logistic regression models, hazardous and problematic drinking habits were associated with nearly 4-fold odds of perpetrating physical IPV with an AOR of 3.78 (95% CI = 1.89, 7.58) and an AOR of 3.56 (95% CI = 1.03, 12.21), respectively (displayed in Table 2). The perpetration of sexual IPV was associated with hazardous alcohol consumption (OR = 2.22, 95% CI = 1.31, 3.78) and dependence symptoms (OR = 5.10, 95% CI = 1.44, 18.07). After controlling for socio-demographic characteristics, food insecurity, and depressive symptoms, the association persisted only for hazardous drinking (AOR = 2.05, 95% CI = 1.22, 3.45). Participants engaging in hazardous drinking demonstrated more than 3-fold odds of reporting past-year perpetration of emotional abuse (AOR = 3.04, CI = 1.79, 5.17). Hazardous drinking (AOR = 1.95, 95% CI = 1.30, 2.91) and dependence symptoms (AOR = 3.69, 95% CI = 1.56, 8.71) increased young men’s odds of perpetrating financial abuse. Perpetration of controlling behaviours was also associated with hazardous drinking after controlling for socio-demographic characteristics, food insecurity, and depressive symptoms (AOR = 2.07, 95% CI = 1.04, 4.10). Four forms of IPV, except physical IPV, were positively associated with the covariate depressive symptoms.

**Table 2.** Crude and adjusted logistic regression models for different drinking patterns and forms of IPV.

Past-year IPV perpetration										
	Physical		Sexual		Emotional		Economic		Controlling	
Variable	OR (95% CI)	AOR (95% CI)	OR (95% CI)	AOR (95% CI)	OR (95% CI)	AOR (95% CI)	OR (95% CI)	AOR (95% CI)	OR (95% CI)	AOR (95% CI)
Past-year alcohol consumption										
Abstainers	Ref	Ref	Ref	Ref	Ref	Ref	Ref	Ref	Ref	Ref
Low-risk drinking	1.1 (0.5, 2.5)	1.1 (0.5, 2.7)	1.1 (0.8, 1.6)	1.1 (0.8, 1.6)	1.11 (0.7, 1.8)	1.1 (0.6, 1.8)	1.4 (0.9, 2.2)	1.3 (0.8, 2.1)	1.2 (0.6, 2.1)	1.0 (0.6, 1.9)
Hazardous drinking	4.0 (2.1, 7.7)*	3.8 (1.9, 7.6)*	2.2 (1.3, 3.8)*	2.1 (1.2, 3.5)*	3.3 (2.0, 5.4)*	3.0 (1.8, 5.2)*	2.1 (1.4, 3.1)*	2.0 (1.3, 2.9)*	2.1 (1.1, 4.1)*	2.1 (1.0, 4.1)*
Problematic drinking	3.7 (1.2, 11.5)*	3.6 (1.0, 12.2)*	2.18 (0.54, 8.75)	1.7 (0.4, 6.9)	1.1 (0.4, 3.2)	0.8 (0.2, 2.9)	1.5 (0.5, 4.8)	1.3 (0.4, 4.2)	1.3 (0.3, 4.9)	1.1 (0.2, 5.2)
Possible dependence	2.6 (0.8, 8.7)	2.5 (0.5, 12.0)	5.10 (1.44, 18.07)*	3.4 (1.0, 11.4)	2.3 (0.7, 7.4)	1.4 (0.4, 4.8)	4.8 (2.0, 11.4)*	3.7 (1.6, 8.7)*	3.7 (0.5, 29.5)	2.4 (0.3, 21.3)
Socio-demographics										
Age, years	-	0.9 (0.8, 1.0)	-	1.0 (0.9, 1.2)	-	1.1 (1.0, 1.2)	-	1.0 (0.9, 1.1)	-	1.1 (1.0, 1.2)*
Education	-	0.7 (0.5, 1.0)*	-	1.1 (0.9, 1.3)	-	1.1 (0.9, 1.4)	-	1.1 (0.9, 1.3)	-	1.6 (1.2, 2.0)*
Employment	-	1.2 (0.7, 2.0)	-	1.2 (0.8, 1.8)	-	0.9 (0.6, 1.3)	-	1.4 (0.9, 2.3)	-	1.0 (0.6, 1.5)
Other predictors										
Food insecurity	-	1.0 (0.6, 1.4)	-	1.5 (1.0, 2.1)*	-	1.2 (0.9, 1.7)	-	1.2 (0.9, 1.5)	-	1.2 (0.9, 1.7)
Depressive symptoms	-	1.2 (0.8, 1.8)	-	1.5 (1.3, 1.8)*	-	1.7 (1.3, 2.1)*	-	1.3 (1.0, 1.6)*	-	1.4 (1.0, 1.9)*

*p < 0.05.

## Discussion

This study found that alcohol use is associated with IPV perpetration among young men in Mwanza, Tanzania, with nuanced findings regarding the role of alcohol use patterns and different forms of IPV.

The prevalence of alcohol consumption (25%) in the sample is consistent with prevalence estimates of other studies among young adults in eastern African countries, including Tanzania [27]. The prevalence of alcohol use was thereby lower than that of young adults in the WHO European Region (44%) and the Region of the Americas (38%) [35]. The prevalence of IPV perpetration reported by this study is similar to the findings from previous studies on IPV perpetration studies in Tanzania and other sub-Saharan countries [36,37]. In a study of men in Dar es Salaam, 20% of the men reported perpetrating emotional IPV, 13% physical IPV, and 28% sexual IPV [36]. In our study, emotional IPV (47%) perpetration and physical IPV (16%) perpetration were higher, whereas sexual IPV (23%) perpetration was lower. Comparisons with controlling behaviour are challenging since this form of IPV has only been measured as an act experienced by women. The prevalence of perpetration reported in this study was far higher with 79% in the past year than that reported by women in the Tanzanian DHS 2015/2016, where controlling behaviours were reported by 34% of 20–24-year-old women [23]. This discrepancy may be explained by the fact that controlling behaviour is an invisible violation that women might not recognise as abusive and therefore find difficult to disclose, while men might not see any stigma attached in reporting it [29,38].

As expected, participants reporting recent alcohol use were likely to report higher rates of perpetration for IPV compared to abstainers. No significant association was found between low-risk alcohol consumption and perpetration of any IPV form. Hazardous drinking was found to be associated with the perpetration of physical, sexual, emotional, and financial IPV and controlling behaviours. Problematic drinking habits predicted only physical IPV, while alcohol dependence symptoms were associated with greater odds of sexual and financial IPV perpetration. Problematic alcohol consumption habits and possible dependence symptoms were associated with a lower number of different forms of alcohol-related IPV than we expected. Although these associations were significant in different models after controlling for important covariates, they demonstrated wide confidence intervals, likely due to their small sample size.

The association between alcohol consumption and the perpetration of different forms of physical, sexual, financial, and emotional IPV among young male adults supports the existing literature on alcohol consumption, especially at higher levels of consumption, as a key predictor of IPV and a major risk factor for IPV [39,40]. As most studies on risk factors of IPV are focused on physical and/or sexual IPV, there is a lack of evidence on the established association between alcohol use and the perpetration of controlling behaviours among men. There is some evidence though that women whose partners engaged in drinking showed higher exposure to controlling behaviours [40]. A study conducted in Vietnam, for example, found that physical and sexual IPV perpetrated by husbands was more severe if it co-occurred with controlling behaviours in comparison to those who only experienced physical or sexual IPV [38].

As our sample included young men between 18 and 24 years of age, our findings cannot be generalised to other age groups. Due to the comparatively low level of alcohol consumption in Mwanza, Tanzania, the results might not be transferable to other regions of the country or elsewhere globally, where rates might be much higher. Given the limitations of the cross-sectional study design, we also could not infer causality between alcohol consumption and perpetration of IPV. Furthermore, the prevalence of problematic drinking habits and IPV are likely underestimated for three reasons. First, levels of alcohol consumption might be underreported since the widespread use of home brews in African countries including Tanzania might not be considered as alcohol use [26]. Second, substance abuse and IPV are sensitive topics, which are difficult to disclose [41]. Specifically, heavy drinkers tend to severely underestimate their drinking patterns [42]. Respondents may underreport both alcohol consumption and IPV due to the social desirability bias, potential social consequences, stigma, or feelings of embarrassment as a result of social norm violations [43]. This might have led to small numbers among those considered to have problematic drinking habits and those engaging in harmful dependence drinking, which in turn is likely to have affected the existence or strength of the association. On the other hand, according to the common assumption, young people are also likely to overreport their drinking habits to be accepted by their peers [42]. Third, our study would have benefitted from additional information on alcohol consumption of the female partner as joint alcohol consumption is known to lead to more heavy drinking and easier escalation of relationship conflict and violence [44].

Despite these limitations, our study highlights that associations exist between different drinking patterns in young people and alcohol-related IPV perpetration and thereby informs the hypothesis generation for future research. Further research integrating both partners’ alcohol consumption, IPV perpetration, and experience is needed to better understand their interrelation. While the covariate mental health was associated with four forms of IPV, further research on its role as a pathway linking alcohol consumption to IPV among young people would be beneficial. Alcohol consumption and mental health are, to a significant degree, modifiable risk factors for IPV perpetration and represent great importance for future interventions [45]. Further research on the risk factors for controlling behaviours and their co-occurrence with other forms of IPV is needed, for example, in the form of longitudinal study designs.

## Conclusions

Our study shows that IPV is prevalent among young men who consume alcohol, as they are also more likely to be perpetrators of all five forms of IPV in relationships than abstainers. A deeper investigation showed that hazardous and problematic drinking habits led to increased odds of IPV perpetration, but not among low-risk drinkers. Further research on emotional abuse and controlling behaviours is needed, as they were only associated with one drinking pattern. Our findings show that when developing alcohol-related policies and IPV interventions tailored to young men, it is crucial to have a differentiated view on drinking patterns and IPV.

## Paper context

There is strong evidence for a connection between IPV and alcohol consumption. This study in Tanzania fills a gap in knowledge by exploring this association with different forms of IPV perpetuated by young men as well as the dose effect of alcohol. Interventions to address alcohol usage therefore need to take IPV into account and alcohol use needs to be addressed in IPV interventions.
